# Incisional Negative Pressure Wound Therapy devices applied after Total Ankle Arthroplasty: A Hospital-Based Health Technology Assessment

**DOI:** 10.1371/journal.pone.0322327

**Published:** 2025-04-29

**Authors:** Jamal Atfeh, Pascale Guerre, Emmanuelle Carre, Jean-Luc Besse, Laure Huot

**Affiliations:** 1 Hospices Civils de Lyon, Pôle de Santé Publique, Service d’Evaluation Economique en Santé, Lyon, France; 2 Université Lyon 1, Inserm U1290 Research on Healthcare Performance (RESHAPE), Lyon, France; 3 Université Lyon 1, Laboratoire Parcours Systémique en Santé, Lyon, France; 4 Hospices Civils de Lyon, Pharmacie Centrale, Saint Genis Laval, France; 5 Hospices Civils de Lyon, Hôpital Lyon Sud, Service de Chirurgie Orthopédique et Traumatologie, Pierre-Bénite, France; Southern Medical University Nanfang Hospital, CHINA

## Abstract

**Introduction:**

A portable, single-use incisional Negative Pressure Wound Therapy (iNPWT) device could be used directly on the wound incision following Total Ankle Arthroplasty (TAA). In order to support local decision-making in a French university hospital for the adoption of such device in this indication, a three-dimensional (clinical, economic and organizational) Hospital Based-Health Technology Assessment (HB-HTA) was conducted.

**Materials and methods:**

The HB-HTA was based on: a literature review; results from the PICO-PTC single-site randomized controlled trial (ClinicalTrial.gov identifier NCT03886818); and semi-structured interviews with healthcare professionals.

**Results:**

Two comparative retrospective cohort studies were retrieved from the literature review. They suggested a decreased incidence of wound healing complications with iNPWT compared to standard dressings, although no difference was found in the PICO-PTC study. There was no significant difference in the PICO-PTC study on Medicine-Surgery-Obstetrics hospital costs between the two strategies: €10,639.65 [CI 95% (7,997.45; 17,988.68)] in the iNPWT group versus €9,672.59 [CI 95% (7,966.29; 13,393.65)] in the standard dressings group. Adoption of this prophylactic strategy would result in an approval to pay a 1.5% supplement to the Medicine-Surgery-Obstetrics hospitalization costs. It could also improve professionals’ workflow, and facilitate wound healing monitoring for nurses in orthopedic departments compared to standard dressings.

**Conclusion:**

The clinical effectiveness of the iNPWT single-use portable device could not be demonstrated compared to standard dressings for prophylactic use, in terms of the incidence of wound healing complications, in patients scheduled for TAA and not selected on the basis of risk factors. However, as this strategy may bring organizational benefits and has not been associated with significant economic costs, the adoption of iNPWT in orthopedic departments could be made according to the surgeon’s clinical expertise, based for instance on the patient’s risk factors for complications.

## Introduction

Total Ankle Arthroplasty (TAA) is an orthopedic surgical procedure known for its rare but severe postoperative complications. In the early post-operative period, these complications are mostly wound healing problems [1–4] which can lead to Surgical Site Infections (SSI) with incidence between 1.2% and 3.3% according literature [2,4,5]. Standard postoperative wound care consists classically in the application of standard sterile dressings at the end of the procedure, followed by regular dressing repairs [6].

For over 20 years, the use of sub-atmospheric pressure dressings has shown positive outcomes in the healing process of various wounds [7]. This Negative Pressure Wound Therapy (NPWT) technology takes action by application of micromechanical forces on the wound, stimulating cell proliferation and accelerating wound healing [8]. NPWT implements a wet wound-healing environment and decreases bacterial colony counts [9]. This technology has been widely used for curative purpose in open wounds, reconstructive surgery, and chronic wounds [10–12]. Over the last decade, NPWT has been also used as a preventive measure of postoperative complications, on closed incisional wounds [13–15]. This prophylactic approach is called incisional NPWT (iNPWT).

Literature has shown possible clinical benefits of iNPWT in several indications. On the basis of a shortened delay in the wound healing process, Norman et al. [16] suggested that iNPWT probably reduces the incidence of SSI compared to standard dressings. Authors’ conclusions were based on Randomized Controlled Trials (RCT) including, among others, patients undergoing orthopedic surgery, but limited to hip and knee arthroplasties [17–20].

Using iNPWT for wound management process of closed incisions after TAA, assisted by a single-use and portable device, could be a new approach that was investigated in an orthopedic surgery department of a French university hospital. Despite a potential clinical benefit, the required iNPWT device is expensive compared to the dressing usually used. However, the prevention of postoperative complications, particularly SSIs which are known to be a burden on the healthcare system with prolonged hospital lengths of stay and/or hospital readmissions [21], could be cost-saving. The device could also provide more flexibility to healthcare professionals for the management of post-operative surgical ankle wounds in the orthopedic department or the rehabilitation center.

A three-dimensional Hospital-Based Health Technology Assessment (HB-HTA) was performed to evaluate the clinical, economic and organizational impacts of using iNPWT on closed incisional wounds after TAA, in order to support local decision-making.

## Materials and methods

The HB-HTA included three dimensions that have a direct impact on deciding whether to introduce a new medical device at the hospital level:

- Clinical effectiveness: This dimension was assessed from a literature review and results from a pragmatic randomized controlled trial conducted in the orthopedic department of the Lyon university hospital (the PICO-PTC study; ClinicalTrial.gov identifier NCT03886818). The outcome of interest was the incidence of wound healing complications and/or SSIs;
- Economic outcomes: This dimension was assessed from a literature review and results from the economic evaluation conducted as part of the PICO-PTC study;
- Organizational impact: This dimension was assessed through semi-structured interviews with healthcare professionals involved in patients care and familiar with the iNPWT device.

The different sources of data used to explore these three dimensions are exposed bellow.

### Literature review

The PubMed Medline database was searched from inception to April 30^th^ 2024 to assess published clinical studies and economic evaluations about the use of iNPWT following TAA.

The following search terms strategy was used: ((“Arthroplasty, Replacement, Ankle”[Mesh] OR “Total Ankle Arthroplasty” OR “total ankle replacement”) AND (clos* OR incision* OR “surgical wound”[Mesh] OR “surgical wound”[All Fields] OR “wound healing”[Mesh] OR “wound healing”[All Fields])) AND (“Negative-Pressure Wound Therapy”[Mesh] OR “Negative-Pressure Wound Therapy”[All Fields] OR “vacuum assisted closure therapy” OR “Negative Pressure Wound Therapy” OR “Negative Wound Therapy” OR VAC OR PICO OR PREVENA). Non comparative studies were excluded. Studies not published in English or French were excluded. All types of economic evaluations could be included.

### The PICO-PTC study

The PICO-PTC study was a single center, non-blinded randomized clinical trial based on usual practices, designed to evaluate the superiority of iNPWT over standard dressings in reducing delayed wound healing after TAA (primary objective). The trial adhered to the Declaration of Helsinki and approval to conduct this study was obtained from a French Ethics Committee (Comité de Protection des Personnes Nord-Ouest III) on January 30^th^ 2019. All patients were given written information and signed a consent form before inclusion. Patients were included from 25 March 2019–10 February 2020; the study ended on 17 February 2021 with the last patient visit scheduled according to the protocol.

Adults aged 18 years and older, scheduled for TAA, and not selected for risk factors for complications [5,22–24], could be included.. Recruitment was completed during the scheduled pre-operative visit and informed consent was obtained from all patients. A single experienced surgeon performed all TAAs. Patients were randomized prior to surgery either to the iNPWT strategy (PICO^TM^ device, Smith and Nephew, UK) or the standard dressing strategy used in the center (CICAPLAIE^TM^ sterile dressing, Smith and Nephew, UK).

In the iNWPT strategy, PICO^TM^ dressing was directly applied on the closed incision wound up to 7 days postoperatively at 80 mm Hg, and then changed to standard dressings. In the standard dressing strategy, CICAPLAIE^TM^ sterile dressings were directly applied postoperatively. After TAA hospitalization (3–5 days, up to 7 days if hemophiliac), patients were addressed and followed up for about 6 weeks in a dedicated rehabilitation center. Two follow-up visits were also scheduled at postoperative months 4 and 12 according to standard practice of the surgeon.

#### Clinical outcomes of interest for the work of HB-HTA.

The rate of wound healing complications was a secondary outcome in the PICO-PTC study and was defined as the presence of exudate; blister; necrosis; or a wound dehiscence. It was measured at Medicine-Surgery-Obstetrics (MSO) and Follow up Care and Rehabilitation (FCR) hospital discharge, and at 4 and 12 months after TAA intervention (during follow-up visits) in each randomization group. The rate of SSI (superficial, deep or of a surgical site other than the incision site) was also a secondary outcome and was defined according to the criteria of the French National Technical Committee on Nosocomial Infections [25]. As a prosthesis or implant was placed, this outcome was measured up to 12 months after TAA according to these criteria.

#### Economic outcomes.

***Measurement and valuation of resources and costs*:** The cost analysis was conducted from a hospital perspective. Time horizon was set at 12 months. Costs were measured in euros (€) at 2022 price year and no discount rate was applied given the time horizon chosen for the analysis (i.e., 12 months). Direct medical costs were collected at individual level for all participants.

MSO hospital costs for initial TAA and possible readmissions were collected after hospital discharge. Hospital stays were identified and classified per Diagnosis Related Groups (DRG) and valued close to their production costs [26], as the valuation was based on a cost-accounting method. Structural costs were excluded from the valuation.

Due to non-access to FCR hospitalization data, except length of stay, one standardized DRG was identified based on FCR national hospital statistics provided by the French Technical Agency for Information on Hospitalization (*Agence Technique de l’Information sur l’Hospitalisation*, ATIH). A mean hospital cost per day was estimated (mean DRG national cost, provided by the National Cost Study (NCS), divided by the corresponding mean national length of stay), and then applied to the actual length of stay of each patient in the PICO-PTC study.

Hospital medical consultations related to incisional wound management were collected in a patient diary until the end of their participation in the study (one year). As these items could not be valued by their production costs, they were valued on the basis of reimbursement tariffs of the French National Health Insurance according to guidelines [26].

Finally, each patient randomized to the iNWPT arm was assigned the cost of a PICO^TM^ kit consisting of a pump, two sterile dressings, 2 AA lithium batteries and 2x6 detachable fastening strips. If an additional kit was needed in case of failure when applying the dressing, it was taken into account. The PICO™ device was valued based on its purchase price (156 € including taxes). Costs of standard dressings, being the standard of care, were assumed to be included in the MSO and FCR hospital costs and were therefore not subject to additional valuation.

***Consideration of uncertainty:*** Identification of patients with outlier values was performed. If identified, these patients would be included in the base-case analysis but excluded in a scenario analysis to test the impact on costs.

Univariate Deterministic Sensitivity Analysis (DSA) was performed on the following items, which were supposed to have a major impact on total costs: MSO and FCR hospitalization costs. Extreme values around tested parameters were defined by their Bias-Corrected and accelerated (BCa) bootstrap 95% Confidence Intervals (CI). Variation of the cost of the iNPWT device was also tested in the analysis, within a range of -50% to +50% of its purchase price, in order to take into account an evolution of the device’s price, and possibly support the adoption of an alternative portable single-use device available in this indication. One parameter was varied at one time to test the impact on the difference in strategies’ total costs. Results were reported in a Tornado diagram.

***Statistical analysis*:** Descriptive quantitative data were presented using means and standard deviations. Descriptive qualitative data were presented using integer numbers and percentage frequencies.

Non-parametric bootstrap method (R=10 000) was used to adjust the skewness distribution of costs. Costs were therefore presented using means assorted with their BCa bootstrapped 95% CI. Costs were also compared between groups with mean cost differences and their BCa bootstrapped 95% CI: a statistical difference in costs was assessed when CI of the difference did not include zero.

All analyses were performed using R (version 4.2.2) within R Studio software.

### Semi-structured interviews

Semi-structured interviews were conducted with healthcare professionals involved in the wound management process after TAA at Lyon university hospital, in order to assess the organizational impact of the iNPWT strategy. Respondents were familiar with the portable single-use iNPWT device (i.e., PICO™) as well as with the standard of care (i.e., standard dressings) and could be physicians, nurses or pharmacists.

An interview survey was built using criteria assessing the organizational impact of medical innovations retrieved from a literature review [27] (S1 File). The final criteria chosen were relevant within the local organizational process, the technology evaluated and its indication. These criteria also had to be unexplored in the clinical and economic dimensions. Criteria are listed below: 1) “Cooperation” changes in the cooperation with other actors/sectors, in the way medical staff work together, in the knowledge sharing; 2) “Training and knowledge” medical device’s learning curve; 3) “Misuse” major risk of inappropriate use; 4) “Managing” pharmaceutical supply process and storage constraints; 5) “Vigilance” changes in monitoring requirements; 6) “Workflow” changes in dressing repair requirements; 7) “Communication and information” changes in mode, frequency, or content of communication/information; 8) “Patient ergonomics” patient autonomy and privacy.

Respondents were asked to inform their occupation and to answer all questions at least by “Yes”, “No” or “Not Applicable”. Each question explored one criterion of the health technology’s organizational impact and enabled us to adjudicate whether or not the iNPWT device had an impact on the criterion. None of the criteria was weighted.

Respondents could also argue an answer. These additional qualitative data were meant to provide elements for discussion, especially to assess if the technology had a positive or negative impact on the organizational process.

## Results

### Clinical dimension

Nine citations were identified from the literature search. A review of titles and abstracts, and full texts when needed, excluded seven citations that did not fulfill the inclusion criteria: one was an expert opinion on the potential benefit of iNPWT in TAA published in 2010; one was a case series on the use of NPWT for random local flaps published in 2010; one presented general management of soft tissue reconstruction of foot and ankle published in 2013; one was a brief literature review on the general use of iNPWT in orthopedic surgery (which retrieved the expert opinion mentioned above in TAA) published in 2014; one was a report of three cases with delayed wound healing after TAA who required tibialis anterior tendon resection published in 2020; one was a retrospective non-comparative study of iNPWT applied after TAA published in 2020; one was a systematic review on biological, mechanical, and pharmacological options to prevent wound complications after TAA published in 2023 (which retrieved the non-comparative study mentioned above and one comparative study considered for analysis). Two retrospective comparative studies with low level of evidence were therefore finally retrieved and analyzed [28,29].

Matsumoto et al. [28] conducted in 2015 a USA single-center and single-surgeon, before-and-after retrospective cohort study, including patients undergoing TAA between 2009 and 2013. Patients managed with standard dressings between February 2009 and May 2012 (n = 37) were compared to patients managed with an iNPWT device (80 mm Hg for 7 days, PICO™) between June 2012 and August 2013 (n = 37). A total of nine (24%) patients presented a wound healing problem (defined as the presence of a wound dehiscence, eschar, or drainage over 3 weeks after the index surgery) in the control group *versus* one (3%) in the iNPWT group (p=0.014). No significant difference was found in SSI (p=0.615) with two (5%) superficial and one (3%) deep infections in the control group versus one (3%) deep infection in the iNPWT group within 30 days after surgery (defined according to criteria of the Centers for Disease Control and Prevention [30]). The deep infection in the control group occurred in a 55-year-old male whose only risk factor was alcohol use. The deep infection in the iNPWT group occurred in a 72-year-old male who had several risk factors including diabetes mellitus, hypertension, corticosteroid use, rheumatoid arthritis, and low lymphocyte count. The four SSIs were associated with wound healing problems. Application of iNPWT could independently predict absence of wound healing problems (Odds Ratio = 0.10; 95% CI [0.01–0.50]; p = 0.004).

Liu et al. [29] conducted in 2020 a before-and-after, Chinese single-center and single-surgeon retrospective cohort study. A total of 21 patients with sterile dressings between January 2010 and December 2016 was compared to 13 patients managed with iNPWT (75 mm Hg for 7 days, V.A.C. Therapy™, KCI, 3M) from January 2017 to June 2018. Incidence of wound complications (defined as the presence of a wound dehiscence, hematoma, blister, skin necrosis, scabbing or abnormal exudation over 3 weeks after the index surgery) was higher in the control group with four (19%) *versus* one (7.7%) in the iNWPT group, although it was not statistically significant (p=0.34). Only one (4.8%) superficial SSI occurred within 30 days after surgery (also defined according to criteria of the Centers for Disease Control and Prevention [30]) in the control group (p=0.62). Risk factors for this patient were not reported.

The PICO-PTC randomized study was conducted in 2019 and included a total of 48 patients (24 in the iNPWT strategy and 24 in the standard dressing strategy). Baseline patient characteristics are summarized in S2 File. Incidence of wound complications following TAA intervention was the same in both groups (4.2%). In the iNPWT strategy, the patient who presented a wound healing complication also presented one superficial SSI (4.2%) during his FCR hospitalization which progressed to a deep SSI at 12 months. The patient was hemophilic with a BMI of 29.4 kg/m^2^. The patient treated with standard dressings presented a wound healing complication during his FCR hospitalization and required a hospital readmission for total skin graft surgical revision. He had a BMI of 37 kg/m^2^ and presented surgical site risk factors at inclusion (i.e., scars on the incision site, surgical approach issues and previous ankle surgery).

Table 1 summarizes our findings concerning incidence of wound healing complications and/or SSIs, as part of the assessment of the clinical dimension in the HB-HTA report.

**Table 1:** Incidence of wound complications and SSIs in three studies evaluating iNPWT following TAA.

Study	Criteria	iNPWT	Standard dressings	p-value
**Matsumoto et al. 2015 (29)** Retrospective cohort study, (80 mm Hg for 7 days, PICO^TM^ Smith and Nephew)	*N*	37	37	
	*Wound complications n (%)*	1 (3.0%)	9 (24.0%)	0.014
	*SSI n (%)*	1 (3.0%)	3 (8.0%)	NS
**Liu et al. 2020 (30)** Retrospective cohort study (75 mm Hg for 7 days, V.A.C. Therapy™, KCI, 3M)	*N*	13	21	
	*Wound complications n (%)*	1 (7.7%)	4 (19.0%)	NS
	*SSI n (%)*	0 (0.0%)	1 (4.8%)	NS
**PICO-PTC trial** Prospective RCT, (80 mm Hg for 7 days, PICO^TM^ Smith and Nephew)	*N*	24	24	
	*Wound complications n (%)*	1 (4.2%)	1 (4.2%)	NS
	*SSI n (%)*	1 (4.2%)	0 (0.0%)	NS

iNPWT: incisional Negative Pressure Wound Therapy; TAA: Total Ankle Arthroplasty; SSI: Surgical Site Infection; RCT: Randomized Controlled Trial; NS: Non Significant.

### Economic dimension

No economic studies were retrieved in the literature in this indication. The economic assessment was based on the economic evaluation conducted as part of the PICO-PTC study.

#### Base case analysis.

Total healthcare costs were €22,324.57 [CI 95% (19,351.35; 32,244.41)] in the iNPWT group and €21,631.79 [CI 95% (19,277.38; 25,490.46)] in the standard dressing group (see S3 File for individual cost data). There was no significant difference in costs between the two groups (Table 2).

**Table 2:** Average costs per patient in euro price year 2022 from the hospital perspective in the PICO-PTC study.

Cost expressed as Mean [95% CI]	iNWPT group (n = 24)	Standard dressing group (n = 24)	Difference^*^ in costs
MSO hospitalization	10,639.65 [7,997.45; 17,988.68]	9,672.59 [7,966.29; 13,393.65]	967.07 [-2,804.43; 6,998.94]
FCR hospitalization	11,515.92 [10,688.25; 13,227.58]	11,904.52 [10,857.14; 14,884.57]	-388.6 [-3,056.46; 1,266.62]
Readmissions	0 [0; 0]	52.6 [0; 157.79]	-52.6 [-274.41; 0]
Dressings	169 [156; 188.5]	0^**^ [0; 0]	169 [156; 195]
Hospital medical consultations	0 [0; 0]	2.08 [0; 6.25]	-2.08 [-11.9; 0]
Overall	22,324.57 [19,351.35; 32,244.41]	21,631.79 [19,277.38; 25,490.46]	692.78 [-3,633.48; 8,603.16]

CI: confidence interval; iNPWT: incisional Negative Pressure Wound Therapy; MSO: Medicine Surgery Obstetrics; FCR: Follow up Care and Rehabilitation.

*Difference in bootstrapped means were computed according to health economics standards (iNPWT – Standard dressing).

**Costs of standard dressings were assumed included in the MSO and FCR hospital costs as they represent the standard of care in this indication.

Main cost drivers were costs of TAA hospitalization in MSO and FCR hospitalization. FCR hospitalization costs were quite similar between the two groups but mean costs were slightly higher for MSO hospitalization in the iNPWT group: €10,639.65 [CI 95% (7,997.45; 17,988.68)] versus €9,672.59 [CI 95% (7,966.29; 13,393.65)] in the control group.

The additional cost of the device represented less than 1% of the total cost when compared to the overall patient trajectory after TAA. From a strict MSO hospital perspective, adoption of an iNPWT device at a purchase price of €156 would result in an approval to pay a 1.5% supplement to the total hospitalization productivity costs of patients undergoing TAA.

#### Sensitivity analyses.

One patient mostly induced higher costs in the iNWPT group. The patient was a 52 years old male hemophilic, with a BMI of 29.4 kg/m^2^, whose overall costs were €74,059.10 with €51,722.90 for MSO and €22,180.20 for FCR hospital costs. He presented a large hematoma on initial hospitalization for TAA, which required additional costly antihemophilic factors due to a prolonged wound healing process. This episode significantly increased the costs of MSO hospitalization. He also presented a wound complication (healing disorder and SSI) which extended his stay in the FCR center. These elements led to consider this patient as an outlier; therefore, the patient was excluded in a scenario analysis. With the outlier excluded, total healthcare costs were lower in the iNWPT group: €20,089.35 [CI 95% (18,578.82; 22,965.27)] versus €21,650.55 [CI 95% (19,295.48; 25,513.28)] (statistically non-significant difference).

**Fig 1:**
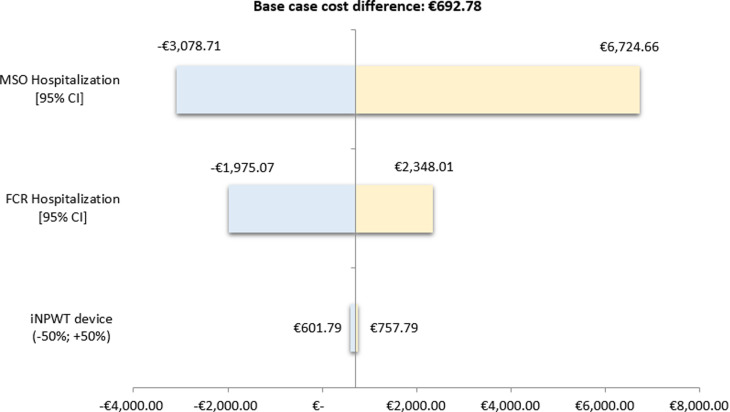
Results of the univariate deterministic sensitivity analysis, Tornado diagram. MSO: Medicine Surgery Obstetrics; FCR: Follow up Care and Rehabilitation; iNPWT: incisional Negative Pressure Wound Therapy.

Uncertainty on economic outcomes was mainly driven by MSO and FCR hospitalizations (Fig 1). Results from the univariate DSA showed that MSO hospitalization had the highest impact on the cost difference between the two strategies, varying from –€3,078.71 (iNPWT being the less costly strategy) to €6,724.66. In contrast, the purchase price of the device had a minor impact on the cost difference (€601.79 to €757.79).

### Organizational dimension

Six semi-structured interviews were conducted. Respondents were: a surgeon and three nurses from the orthopedic surgery department; a pharmacist in charge of the Medical Devices supply; and a physician from the FCR center. Organizational criteria impacted were “Workflow”, “Cooperation”, “Communication and information”, “Vigilance”, and “Patient ergonomics” according to the healthcare professionals (Table 3).

**Table 3:** Organizational impacts of the portable single-use iNPWT device as reported by healthcare professionals.

Criteria Professional	MSO *Surgeon*	FCR *Physician*	MSO *Pharmacist*	MSO *Nurse 1*	MSO *Nurse 2*	MSO *Nurse 3*	Total
WORKFLOW	yes^a^	yes	n/a^c^	yes	yes	yes	**5/5**
COOPERATION	yes	yes	yes	no	no	no	**3/6**
COMMUNICATION AND INFORMATION	yes	no	yes	yes	no	no	**3/6**
VIGILANCE	no^b^	yes	n/a	yes	no	no	**2/5**
PATIENT ERGONOMICS	no	yes	n/a	yes	no	no	**2/5**
MISUSE	no	no	no	n/a	n/a	n/a	**0/3**
TRAINING AND KNOWLEDGE	no	no	no	no	no	no	**0/6**
MANAGING	no	no	no	no	no	no	**0/6**

iNPWT: incisional Negative Pressure Wound Therapy; MSO: Medicine-Surgery-Obstetrics; FCR: Follow up Care and Rehabilitation.

a yes: The iNPWT device impacted the criterion; ^b^ no: The iNPWT device did not impact the criterion; ^c^ n/a: Not applicable for the respondent.

The main positive organizational impact of the iNPWT device was improvement of professionals’ workflow as it reduced frequency of dressing repairs when compared to standard dressings. However, the surgeon highlighted that repair was a bit more delicate as he had to manage the change of the iNPWT himself at the bed side, which took around 10 minutes, versus 3–4 minutes for standard dressings (their change being managed by nurses, and only controlled by the surgeon).

The physicians and the pharmacist pointed out changes in the cooperation between professionals. For them, this criterion was impacted as the iNPWT device required continuous knowledge sharing on the technology (alarm system, dressing leakage, etc.) due to turnover of nurses in the department, and internal reorganization for the discharge of patients to the FCR center. However, none of the nurses indicated that the iNPWT device had any impact on cooperation.

In addition to patient oral information given by the surgeon, the pharmacist created an informational brochure delivered to the patient. In routine, patient oral information could be delegated to nurses. In the orthopedic surgery department, the patient was also an actor of his wound’s monitoring: he was given complementary information on proper functioning of the iNPWT device so he could warn nurses of a default. For example, a loud noise could inform on a dressing’s poor sealing.

Concerning vigilance requirements, answers were heterogeneous. The surgeon and two nurses indicated that iNPWT did not change the frequency of dressings’ monitoring. The nurses also reported that iNPWT dressings had a positive impact for wound healing monitoring, as it required less nursing staff: with standard dressings, nurses had to open all patients’ dressings of the orthopedic department in the morning so that the surgeon could examine the wound and give care instructions. Due to its occlusive nature, monitoring was facilitated with iNPWT requiring only monitoring of blood saturation of the dressing. On the other hand, the FCR physician and one nurse indicated that frequency of monitoring was increased during their first uses of the device, especially to quickly check if the device was still active and that seal was maintained.

Only the FCR physician and one nurse assumed that the iNPWT device could improve patient’s autonomy and privacy. The surgeon, the FCR physician and the pharmacist indicated that there was no major risk of inappropriate use. The learning curve and the pharmaceutical supply were not a barrier for the device’s routine use after TAA.

## Discussion

To our knowledge, this is the first HB-HTA report evaluating clinical, economic and organizational dimensions of a portable single-use iNPWT device applied prophylactically on closed incisions following TAA. It relied also on the first economic evaluation based on a RCT available in this indication.

Incidence of wound complications and/or SSIs was the clinically relevant outcome in this indication. However, the results were heterogeneous among the studies assessed, definition of outcomes slightly differed and available data was of low-level of evidence. The only RCT conducted in this indication did not show a statistically significant difference in the incidence of wound complications between iNPWT and standard dressing for unselected patients undergoing TAA (it should be noted that these were secondary outcomes of the study).

The main findings of the economic assessment were the absence of significant difference in costs between the two strategies from a hospital perspective. The purchase price of the device represented less than 1% of the total cost when compared to the mean total healthcare cost in the iNPWT strategy.

Finally, adoption of a single use and portable iNPWT device could have a positive impact on the organizational process of MSO hospitals and FCR centers compared to standard dressings following TAA. It could especially improve professionals’ workflow with a reduced frequency of dressing repairs, and enable less nursing staff requirements for wound healing monitoring.

The PICO-PTC study design was based on local clinical practices. One single experienced surgeon performed all TAA procedures and prophylactically applied the iNPWT device for each patient randomized to the corresponding strategy. It also followed the actual pathway of patients managed by the orthopedic surgery department involved: follow up was conducted according to routine care with patients addressed to the standardized FCR center, with a staff accustomed to monitor these patients. Therefore, this pragmatic trial could be used to supply a HB-HTA at the Lyon university hospital, and provide accurate information for hospital stakeholders with clinical and economic data close to real world clinical practice [31].

However, a larger sample size trial, with a Number Needed to Treat computed on the basis of incidence of complications in this indication could be needed to demonstrate a clinical benefit of iNPWT compared to standard dressings, and to efficiently supply the clinical dimension of the HB-HTA report. A USA randomized controlled trial (ClinicalTrial.gov identifier NCT05064696) with wound complications as the primary endpoint and an estimated enrolment of 150 participants is currently ongoing, and could provide an answer in the near future.

On the other hand, the economic assessment suggested that adopting the iNPWT device in this indication would result in an approval to pay a 1.5% supplement to the MSO hospitalization productivity costs of patients undergoing TAA. This estimation, solely focused on a budgetary approach, could be informative for hospital decision makers, as no economic evaluation of iNPWT following TAA has been retrieved in the literature.

Adding organizational criteria enriches the HB-HTA and is strongly recommended [32]. Medical devices represent a wide and heterogeneous group of products and their purpose is sometimes not fully therapeutic: some may be designed to increase the quality of life of patients or intended for health professionals to improve patients’ management. Integration of non-clinical criteria is therefore relevant for a better assessment of these health products [33]. The organizational assessment although does have limitations as the surveys were built and the interviews conducted retrospectively. Respondents were therefore exposed to memory bias and some answers could have been biased due to knowledge of the study results (response bias). These organizational criteria could, in the future, be thought from the protocol design stage of a clinical trial, as assessment of this scope becomes now essential for healthcare systems.

This HB-HTA could have also considered the patient’s perspective [34,35]. This is especially relevant for this type of medical device, designed to improve patient ergonomics considering its size and portability, and as the patient was an actor of the wound healing monitoring. However, it was not scheduled before the end of the study. Interviewing health professionals was still relevant as this new prophylactic approach is likely to reshape wound management practices in orthopedic departments or rehabilitation centers.

In conclusion, this work was useful in helping to make decisions regarding the use of the iNPWT single-use portable device for prophylactic purpose in TAA. The clinical effectiveness of this strategy compared to standard dressings could not be demonstrated, in terms of the incidence of wound healing complications, in patients scheduled for TAA and not selected on the basis of risk factors. However, as this strategy may bring organizational benefits and has not been associated with significant economic costs, the adoption of iNPWT in orthopedic departments could be made according to the surgeon’s clinical expertise, based for instance on the patient’s risk factors for complications.

## Supporting information

S1 File Interview protocol for the organizational assessment. (DOCX)

S2 File Baseline patient characteristics in the PICO-PTC study. (DOCX)

S3 File Individual cost data. (XLSX)
