# Supplementary material for: Incisional Negative Pressure Wound Therapy devices applied after Total Ankle Arthroplasty: A Hospital-Based Health Technology Assessment
Source: PLoS One. 2025 Apr 29;20(4):e0322327. doi: 10.1371/journal.pone.0322327 (PMC12040113; doi:10.1371/journal.pone.0322327)
Supplement: S1 File — (DOCX) [file pone.0322327.s001.docx]

Organizational assessment

Interview protocol

The aim is to evaluate the organizational impact of using an incisional Negative Pressure Wound Therapy (iNPWT) device on closed incisional wounds after Total Ankle Arthroplasty (TAA).

Please answer at least “Yes”, “No”, or “Not applicable”.

Thanks for your collaboration

The Health Economics Evaluation Department

1. What is your occupation?

☐ Nurse

☐ Pharmacist

☐ Orthopedic surgeon

☐ Other (specify) : ………………………….

1. Does the iNPWT device significantly change cooperation between health professionals for the management of the post-TAA wound *(knowledge sharing on the technology, reorganization of health professionals’ schedules etc.)*?

☐ Yes

☐ No

☐ Not Applicable

Additional comments:

…………………………………………………………………………………………………...

…………………………………………………………………………………………………...

…………………………………………………………………………………………………...

1. In your opinion, could the iNPWT device’s learning curve be a barrier to its routine use following TAA?

☐ Yes

☐ No

☐ Not Applicable

Additional comments:

…………………………………………………………………………………………………...

…………………………………………………………………………………………………...

…………………………………………………………………………………………………...

1. In your opinion, is there a major risk of inappropriate use of the iNPWT device *(dressing application, connection to the aspirating system, etc.)* that could alter the wound management process?

☐ Yes

☐ No

☐ Not Applicable

Additional comments:

…………………………………………………………………………………………………...

…………………………………………………………………………………………………...

…………………………………………………………………………………………………...

1. Could the pharmaceutical supply process of the iNPWT device and its consumables in the healthcare service, be a barrier to its routine use (*storage limitations, communication with hospital pharmacy etc.*)?

☐ Yes

☐ No

☐ Not Applicable

Additional comments:

…………………………………………………………………………………………………...

…………………………………………………………………………………………………...

…………………………………………………………………………………………………...

1. Does the iNPWT device change monitoring requirements of the post-TAA wound compared to standard dressings?

☐ Yes, monitoring requirements increased

☐ Yes, monitoring requirements decreased

☐ No

☐ Not Applicable

Additional comments:

…………………………………………………………………………………………………...

…………………………………………………………………………………………………...

…………………………………………………………………………………………………...

1. Does the iNPWT device change dressing repair requirements of the post-TAA wound compared to standard dressings?

☐ Yes, dressings repair requirements increased

☐ Yes, dressings repair requirements decreased

☐ No

☐ Not Applicable

Additional comments:

…………………………………………………………………………………………………...

…………………………………………………………………………………………………...

…………………………………………………………………………………………………...

1. Does the iNPWT device require communication of additional patient information *(how to use the device, patient brochure, etc.)*?

☐ Yes

☐ No

☐ Not Applicable

Additional comments:

…………………………………………………………………………………………………...

…………………………………………………………………………………………………...

…………………………………………………………………………………………………...

1. In your opinion, does the iNPWT device offer greater patient autonomy and privacy for the management of his or her post-TAA wound *(personal hygiene, private life etc.)*?

☐ Yes

☐ No

☐ Not Applicable

Additional comments:

…………………………………………………………………………………………………...

…………………………………………………………………………………………………...

…………………………………………………………………………………………………...
