# Supplementary material for: Incisional Negative Pressure Wound Therapy devices applied after Total Ankle Arthroplasty: A Hospital-Based Health Technology Assessment
Source: PLoS One. 2025 Apr 29;20(4):e0322327. doi: 10.1371/journal.pone.0322327 (PMC12040113; doi:10.1371/journal.pone.0322327)
Supplement: S2 File — (DOCX) [file pone.0322327.s002.docx]

Baseline patient characteristics in the PICO-PTC study

| **Study population** | | **Intervention group**  (n = 24) | **Control group**  (n = 24) | **Total**  (n = 48) |
| --- | --- | --- | --- | --- |
| Age (years), mean (sd) | | 60.5 (14) | 61.8 (14) | 61.2 (13.8) |
| Age < 65 years, n (%) | | 12 (50.0) | 12 (50.0) | 24 (50.0) |
| Sex, n (%) | |  |  |  |
|  | *Male* | 19 (79.2) | 16 (66.7) | 35 (72.9) |
|  | *Female* | 5 (20.8) | 8 (33.3) | 13 (27.1) |
| BMI (kg/m^2^), mean (sd) | | 27.1 (3.8) | 25.8 (3.8) | 26.5 (3.8) |
| BMI >30 kg/m², n (%) | | 5 (20.8) | 3 (12.5) | 8 (16.7) |
| Surgical site risk factors^*^, n (%) | |  |  |  |
|  | *Yes* | 12 (50) | 10 (41.7) | 22 (45.8) |
|  | *No* | 12 (50) | 14 (58.3) | 26 (54.2) |
| Type 2 diabetes, n (%) | | 1 (4.2) | 1 (4.2) | 2 (4.2) |

sd: standard deviation; BMI: Body Mass Index

^*^ Scars on the incision site, surgical approach issues or previous ankle surgeries
